# Neuroprotective Effect of Ceftriaxone on MPTP-Induced Parkinson's Disease Mouse Model by Regulating Inflammation and Intestinal Microbiota

**DOI:** 10.1155/2021/9424582

**Published:** 2021-12-13

**Authors:** Xiaoting Zhou, Jiachen Lu, Kehong Wei, Jing Wei, Puyuan Tian, Mengyun Yue, Yun Wang, Daojun Hong, Fangjun Li, Bo Wang, Tingtao Chen, Xin Fang

**Affiliations:** ^1^Department of Neurology, The First Affiliated Hospital of Nanchang University, Nanchang, Jiangxi 330006, China; ^2^Department of Neurology, Qingyuan People's Hospital, The Sixth Affiliated Hospital of Guangzhou Medical University, Qingyuan, Guangdong 511500, China; ^3^National Engineering Research Center for Bioengineering Drugs and the Technologies, Institute of Translational Medicine, Nanchang University, Nanchang, Jiangxi 330031, China

## Abstract

Parkinson's disease (PD) is a common degenerative disease of the central nervous system. Although some drugs can alleviate the progress of PD, their long-term use will lead to complications, so it is still necessary to find new drugs to delay or cure PD effectively. In view of the difficulty in developing new drugs, it is imperative to discover new functions of existing compounds that could be used to treat PD. In this study, 1-methyl-4-phenyl-1,2,3,6-tetrahydropyridine (MPTP) was used to induce PD symptoms in a mouse model. Subsequently, these mice were treated with the antibiotic ceftriaxone. Ceftriaxone alleviated the behavioural and neuropathological changes induced by MPTP, downregulated the expression of glial fibrillary acidic protein (GFAP) and ionised calcium-binding adapter molecule 1 (Iba1) as markers of astroglia and microglia, respectively, and reduced the expression of neuroinflammation-related Toll-like receptor 4 (TLR4), myeloid differentiation primary response 88 (MyD88), and phosphorylated nuclear factor kappa-B (p-NF-*κ*B)/NF-*κ*B in the brain of PD mice. In addition, ceftriaxone reduced the abundance of pathogenic bacteria of the genus *Proteus* and increased the abundance of probiotic *Akkermansia*. Finally, ceftriaxone treatment increased the expression of the tight junction proteins zona occludens-1(ZO-1) and occludin in the colon, decreased the expression of the inflammation-related proteins TLR4, MyD88, and NF-*κ*B in the colon, and decreased the serum concentration of the proinflammatory cytokines interleukin-1*β* (IL-1*β*), IL-6, and tumour necrosis factor-*α* (TNF-*α*). These results indicate that ceftriaxone had a neuroprotective effect on MPTP-induced PD mice, and its neuroprotective effect could be through regulating inflammation and intestinal microbiota. While we showed that ceftriaxone exerts a neuroprotective effect in an MPTP-induced PD mouse model, our findings are limited to the short-term effects of ceftriaxone. Additional work using transgenic mice is required to determine the long-term effects of ceftriaxone. In addition, the dose and frequency of ceftriaxone use should be evaluated.

## 1. Introduction

Parkinson's disease (PD) is a complex and progressively degenerative disease of the central nervous system; it affects more than 6 million people throughout the world [1]. The clinical features of PD include motor and nonmotor symptoms [2]. These symptoms severely affect the quality of life of patients and bring great burden to society [3]. The main pathologies of PD are the loss of dopaminergic neurons in the substantia nigra pars compacta (SNc) of the midbrain and the accumulation of *α*-synuclein in the cytoplasm of neurons form Lewy bodies. The aetiology of PD is not clear but may be related to various risk factors such as excitotoxicity, oxidative stress, and neuroinflammation. The current treatment of PD includes levodopa, dopamine agonists, monoamine oxidase (MAO) inhibitors, anticholinergics, and amantadine, all of which can improve symptoms and slow disease progression. However, none of these treatments can completely cure the disease, and there are some side effects [4]. Although new treatments such as glutamate receptor antagonists, antiapoptotic agents, and antioxidants have been proposed—with the potential to protect nerve cells and slow the progression of PD—there is a lack of scientific evidence to support these claims in clinical practice [5].

Increasing evidence suggests there is bidirectional communication between the gut and the brain [6–8]. Patients with PD have an intestinal microbiota disorder, which is specifically manifested in the increased abundance of *Lactobacillus* and *Enterobacteriaceae* and the decreased abundance of *Prevotella*, *Clostridium coccoides*, and *Bacteroides fragilis* [9, 10]. Recently, GV-971 was approved for the treatment of Alzheimer's disease (AD) by the National Medical Products Administration for its ability to remodel the intestinal microbiota. Targeting the intestinal microbiota for the treatment of AD opens up a new way to treat other neurological diseases [11].

The use of antibiotics has a long-term impact on the composition and diversity of the intestinal microbiota, and it has been reported that the use of antibiotics—especially macrolides and lincosamides—is related to the increased risk of PD [12]. Nevertheless, some antibiotics, such as ceftriaxone, have been reported to have neuroprotective properties that are unrelated to their antibacterial activity [13]. Rothstein et al. [13] first reported that the *β*-lactam antibiotics (such as ceftriaxone) have a neuroprotective effect on ischaemic brain injury. Subsequent studies have proved that ceftriaxone exerts a neuroprotective effect in various neurodegenerative diseases, such as AD [14], PD [15], amyotrophic lateral sclerosis (ALS) [16], and Huntington's disease (HD) [17].

Although ceftriaxone has been used commonly in the clinic, little work has been done to explore its potential mechanisms of adjusting intestinal microorganisms in PD. In this study, ceftriaxone was injected intraperitoneally into a mouse model of PD, induced by 1-methyl-4-phenyl-1,2,3,6-tetrahydropyridine (MPTP), and the neuroprotective effect of ceftriaxone in these mice was explored using behavioural tests, immunofluorescence, Western blotting, and high-throughput 16S ribosomal DNA (rDNA) sequencing.

## 2. Materials and Methods

### 2.1. Animals

The mice used in this experiment were purchased from SJA Laboratory Animal Co. (Changsha, Hunan, China). All mice were male C57BL/6 and weighed 25-30 g. The purchased mice were allowed to adapt to the laboratory environment for 1 week before the experiment. The mice were kept on a 12-h photoperiod, with a temperature of 22 ± 2°C and humidity of 50% ± 15%. Laboratory food and water were available *ad libitum*.

The study had been approved by the Laboratory Animal Ethics Committee of Nanchang Royo Biotech Co., Ltd. (license No. RYE2019041502) on 15 April 2019, and all experiments were conducted according to the established guidelines.

### 2.2. Experimental Design and Treatment

**Figure 1:**
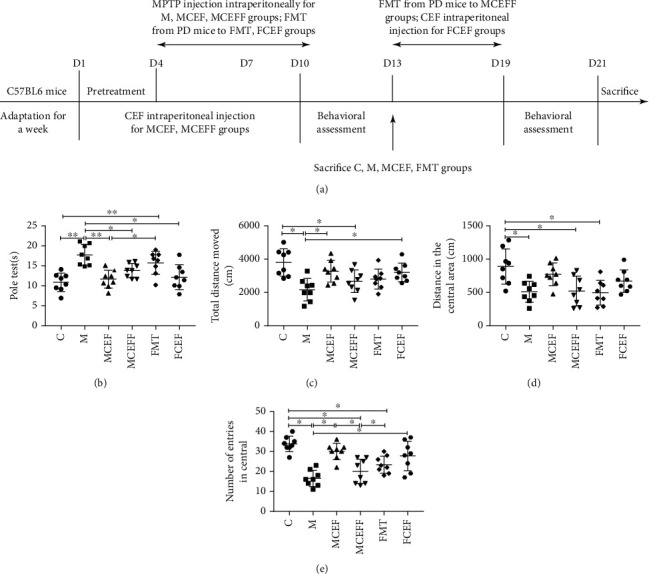
Ceftriaxone improved MPTP-induced motor dysfunction in PD mice. (a) Treatment schedule to explain the design of the whole experimental. (b) Ceftriaxone improved the bradykinesia mice induced by MPTP (pole test). (c) Ceftriaxone increased the total distance moved for PD mice (open-field test). (d) Ceftriaxone increased the distance in the central area moved for PD mice (open-field test). (e) Ceftriaxone increased the number of entries in central (open-field test). C group (*N* = 8), control group; M group (*N* = 8), MPTP group; MCEF group (*N* = 8), MPTP + ceftriaxone group; MCEFF group (*N* = 8), MPTP + ceftriaxone + fecal microbiota transplantation group; FMT group (*N* = 8), fecal microbiota transplantation group, FCEF group (*N* = 8), fecal microbiota transplantation group + ceftriaxone group. Data are presented as means ± SD. One-way repeated-measures ANOVA with Tukey's test for multiple comparisons (b–e, respectively); ^∗^*p* < 0.05, ^∗∗^*p* < 0.01. CEF: ceftriaxone; MPTP: 1-methyl-4-phenyl-1, 2, 3, 6-tetra-hydropyridine.

After 1 week of adaptation, the mice were divided into six groups, each with 15 mic. (i) A control group received saline (C group). (ii) A group was given a daily intraperitoneal injection of MPTP (Sigma, Cat# M-0896) of 20 mg/kg for 7 days to establish a PD mouse model (M group). (iii) A group was treated with ceftriaxone (200 mg/kg, once a day, intraperitoneal injection) for 7 days while using MPTP to establish a PD mouse model (MCEF group). (iv) A group was treated with ceftriaxone (200 mg/kg, once a day, intraperitoneal injection) for 7 days while using MPTP to establish a PD mouse model. Behaviour was evaluated in these mice. Then, the mice were transplanted with 200 *μ*l of faecal microbiota (from PD mice), containing 1 × 10^7^ colony-forming units (CFU)/ml every day for 7 days (MCEFF group). (v) A group received faecal microbiota transplantation from PD mice, namely, 200 *μ*l (microbial content of 1 × 10^7^ CFU/ml) intragastric gavage per day for 7 days (FMT group). (vi) A group received faecal microbiota transplantation from PD mice, namely, 200 *μ*l (microbial content of 10^7^ CFU/ml) gavage per day for 7 days. Behaviour was evaluated in these mice, followed by ceftriaxone administration (200 mg/kg, once a day, intraperitoneal injection) for 7 consecutive days (FCEF group). As shown in Figure 1, we performed behavioural tests on all mice on days 7-10, and euthanised mice in groups C, M, MCEF, and FMT. Then, the MCEFF group was transplanted with faecal bacteria, and the FCEF group was treated with ceftriaxone, and behavioural tests were performed on these mice on days 16-19. After the test, all mice were euthanised. Five mice were intracardially perfused with 30 ml of saline and then injected with 100 ml of 4% (*v*/*v*) paraformaldehyde (Sigma-Aldrich, Cat# P6148) prepared in phosphate-buffered saline (PBS). The entire perfusion process was completed within 5 min, and the perfusate was injected with a 50 ml syringe. The brain was collected immediately and then fixed in 4% (*v*/*v*) paraformaldehyde for immunofluorescence. The brains and colon tissues of the remaining mice were collected, quickly frozen in liquid nitrogen, and stored at -80°C for future molecular biology experiments (Figure 1(a)).

### 2.3. Preparation of Faecal Microbiota from PD Mice

In this experiment, the donors of faecal microbiota transplantation were PD mice in the M group, and faeces of mice were collected after daily intraperitoneal injection of MPTP for 5-6 days. One or two grams of faeces were taken from the collected faeces and mixed with 1 ml of sterile saline, and then, the supernatant was filtered and counted on the plate after multiple dilutions. One gram of faeces contained 1 × 10^9^ to 1 × 10^10^ CFU of microbes; faeces were collected according to the calculated number of faecal microbiota. The faeces of mice were weighed and added to about 5-times the volume of sterile saline. After mixing, the faeces were added to saline (final volume of 50 ml) and oscillated in an oscillator for 2 min for full mixing. Faeces were placed at room temperature for 30 min, the supernatant was collected and centrifuged, and then, the precipitate was collected. The precipitate was washed twice with sterile normal saline, and the final precipitate was added to the appropriate volume of normal saline and mixed well. Finally, the preparation was given to the mice intragastrically.

### 2.4. Behavioural Testing

The pole test is often used to assess the motor coordination in PD mice. A metal rod with a diameter of 1 cm and a length of 50 cm was wrapped with bandage gauze to prevent the mice from slipping, and the bottom of the metal rod was placed in a cage. The mouse was placed head down on the top of the rod. The time it took the mouse to return to the cage freely was recorded, ending when the hind limbs of the mouse reached the bottom of the cage. Before the formal test, the mice were trained for 2 days. Each mouse was tested three times, with 15 min between trials. Finally, we take the average of the three trials for analysis.

The open-field test was used to detect behavioural changes of the mice—such as exploration—in the new environment. The open-field test was performed in a square, with a length, width, and height of 50 cm × 50 cm × 40 cm, and black perimeter walls. It was divided into 25 squares with equal area, which were then divided into the edge area and the central area (the four small squares in the centre). During the experiment, mice were put into a corner of the arena, and their free movement over 10 min was recorded with a camera. At the end of the experiment, the arena was swabbed with 75% alcohol to avoid the next mouse from disturbing by the left scent.

### 2.5. Immunofluorescence

The fixed brains were embedded in paraffin. The tissue was then sectioned, and the sections were dewaxed to water, subjected to antigen retrieval, and incubated with bovine serum albumin (BSA) to block nonspecific protein binding. The tissues were incubated overnight at 4°C with the following primary antibodies: rabbit antityrosine hydroxylase (TH; 1 : 200; Proteintech, Cat# 25859-1-AP), rabbit antiglial fibrillary acidic protein (GFAP; 1 : 200; Proteintech; Cat# 16825-1-AP), and rabbit anti-ionised calcium-binding adapter molecule 1 (Iba1; 1 : 100; Abcam; Cat# ab178847). Subsequently, the tissues were washed on a decolourisation shaker with PBS and then incubated with the corresponding secondary antibody at room temperature for 50 min. After mounting the sections, they were observed under a fluorescence microscope and images captured.

### 2.6. Western Blotting

The brain and colon tissues were homogenised to extract proteins. The protein concentration was determined by using the BCA protein quantitative kit, and the protein was separated by polyacrylamide gel electrophoresis using 10%-12% gels. The electrophoresis time was adjusted according to the molecular weight of the protein of interest. After electrophoresis, the separated protein was transferred to polyvinylidene fluoride membrane. The membrane was incubated with 5% nonfat milk in Tris-buffered saline with Tween 20 (TBST) at room temperature for 1-2 h to block nonspecific protein binding. The membrane was then incubated overnight at 4°C with one of the following antibodies: rabbit anti-TH (1 : 2000; Proteintech, Cat# 25859-1-AP), rabbit antiexcitatory amino acid transporter 2 (EAAT2, GLT-1; 1 : 2000; Proteintech; Cat# 22515-1-AP), rabbit anti-*α*-synuclein (*α*-syn; 1 : 1000; Cell Signaling Technology; Cat# 4179S), rabbit anti-brain-derived neurotrophic factor (BDNF; 1 : 5000; Abcam; Cat# ab108319), rabbit antiglial cell line-derived neurotrophic factor (GDNF; 1 : 5000; Abcam; Cat# ab176564), rabbit anti-GFAP (1 : 10000; Proteintech; Cat# 16825-1-AP), rabbit anti-Iba1 (1 : 1000; Abcam; Cat# ab178847), mouse anti-Toll-like receptor 4 (TLR4; 1 : 1000; Santa Cruz Biotechnology, Cat# sc-293072), rabbit antimyeloid differentiation primary response gene 88 (MyD88; 1 : 1000; Proteintech; Cat# 23230-1-AP), rabbit antiphosphorylated-p65 (p-p65; 1 : 1000; Abcam; Cat# ab86299), rabbit anti-p65 (1 : 1000; Cell Signaling Technology; Cat# 8242S), rabbit anti-zona occludens 1 (ZO-1; 1 : 5000; Proteintech; Cat# 21773-1-AP), rabbit antioccludin (1 : 1000; Proteintech; Cat# 13409-1-AP), and rabbit anti-*β*-actin (1 : 1000; Cell Signaling Technology; Cat# 4970S). The membrane was washed with TBST and then incubated with the appropriate secondary antibody at room temperature for 1-2 h. The enhanced chemiluminescence detection system (Pierce) was used to observe the membranes. The ImageJ software (National Institutes of Health) was used to calculate the density determination.

### 2.7. Serum Cytokine Measurement

Blood (100-150 *μ*l) was collected after anaesthesia. The blood was incubated at 4°C for 30 min and then centrifuged at 1500 g for 15 min; the upper serum was collected and stored at -80°C. The levels of several cytokines were determined by using commercially available enzyme-linked immunosorbent assay (ELISA) kits according to manufacturer's protocols: IL-6 (Proteintech, Cat# KE10007), TNF-*α* (Proteintech, Cat# KE10002), and IL-1*β* (Proteintech, Cat# KE10003).

### 2.8. DNA Extraction

Faecal samples from groups C (*N* = 8), M (*N* = 8), MCEF (*N* = 8), MCEFF (*N* = 8), FMT (*N* = 8), and FCEF (*N* = 8) were collected after the behavioural testing, and the TIANamp Bacteria DNA Kit (TianGen) was used to extract microbiota genomic DNA. The extracted DNA was quantified by using a nanodrop spectrophotometer, and the quality of DNA extraction was visualised by using 1.2% agarose gel electrophoresis. The 16S rDNA V4 region was amplified using primers 515F (5′-GTGCCAGCMGCCGCGGTAA-3′) and 806R (5′-GGACTACVSGGGTATCTAAT-3′). Then, the polymerase chain reaction (PCR) products were sequenced on the IlluminaHiSeq 2000 platform (Illumina, Inc.) (GenBank accession number PRJNA659569).

### 2.9. High-Throughput 16S rDNA Amplicon Sequencing Analysis

The Illumina MiSeq/NovaSeq platform was used to conduct paired-end sequencing of community DNA fragments. The analysis software QIIME2 (version 2019.4, https://docs.qiime2.org/2019.4/tutorials/) and the QIIME2 dada2 analysis process were used. Sequences were quality filtered, denoised, and merged, and chimera were removed to obtain amplicon sequence variants (ASVs). In addition, the Vsearch software analysis process was used for quality filtering, merging, chimera removal, and clustering to obtain operational taxonomic units (OTUs) [18]. According to the distribution of ASV/OTU in different samples, the *α*-diversity level of each sample was evaluated.

The Chao1 index estimates the number of species that actually exist in the community by calculating the ASV/OTU that are only detected once and twice in the community. The Faith_pd index evaluates the degree of genetic diversity of the community by calculating the full length of the clade occupied by the ASV/OTU representative sequence in the sample in the phylogenetic tree constructed by it. The *β*-diversity index represents the difference between samples. Principal coordinate analysis (PCoA), nonmetric multidimensional scaling (NMDS), and other nonconstrained sorting methods were used to reduce the dimensionality of multidimensional microbial data and to display the distribution of samples on the continuous sort axis to show the main trend of the data changes. PCoA expands the sample distance matrix in a low-dimensional space after projecting it and preserves the distance relationship of the original sample to the utmost extent.

### 2.10. Data Analysis

Prism version 7.0 (GraphPad Software, San Diego, CA, USA) was used for data analysis. Statistical analysis was conducted by one-way analysis of variance (ANOVA) followed by Tukey's test for multiple comparisons, as indicated in the figure legends. The data are presented as mean ± standard deviation (SD), and *p* < 0.05 was regarded to be statistically significant.

## 3. Results

### 3.1. Ceftriaxone Improved the Motor Dysfunction and Decreased Exploratory Ability Caused by MPTP

To investigate the effects of ceftriaxone on PD mice, we established a subacute PD mouse model using MPTP and then conducted the faecal microbiota transplantation experiment (Figure 1(a)). In the pole test, MPTP-treated mice showed significant motor dysfunction compared with the control group (M vs.C = 17.72 s vs.10.89 s; *p* < 0.001), and ceftriaxone significantly alleviated MPTP-induced motor dysfunction (MCEF vs.M = 11.67 s vs.17.72 s; *p* < 0.001), and motor dysfunction recurred in the MCEFF group, which underwent transplantation of faecal microbiota from PD mice (MCEFF vs.MCEF = 13.83 s vs.11.67 s) (Figure 1(b)). In addition, when we transplanted the faecal microbiota of PD mice to normal mice, the mice in the FMT group showed motor impairment compared with the control group (FMT vs.C = 15.74 s vs 10.89 s; *p* < 0.01). Notably, the use of ceftriaxone could reverse the movement impairment caused by faecal microbiota transplantation from PD mice (FCEF vs.FMT = 12.16 s vs.15.74 s; Figure 1(b)).

In the open field test, mice in the M group showed decreased exploratory ability after injection of MPTP compared with the C group, as indicated by the total movement distance (M vs.C = 2169 cm vs.3806 cm; Figure 1(c)), distance moved in the central area (M vs.C = 510.6 cm vs. 890.7 cm; Figure 1(d)), and the number of entries in the central area (M vs.C = 16.5 vs.33.75; Figure 1(e)) (*p* < 0.001). Additionally, the use of ceftriaxone increased the exploratory ability of MPTP-induced PD mice. In the MCEFF and FMT groups, the transplantation of PD microbiota decreased the exploratory ability of mice, and the use of ceftriaxone reversed the behavioural and mental changes of the mice caused by PD microbiota in the FCEF group (Figures 1(c)–1(e)).

### 3.2. Effect of Ceftriaxone on MPTP-Induced Neuropathological Changes

**Figure 2:**
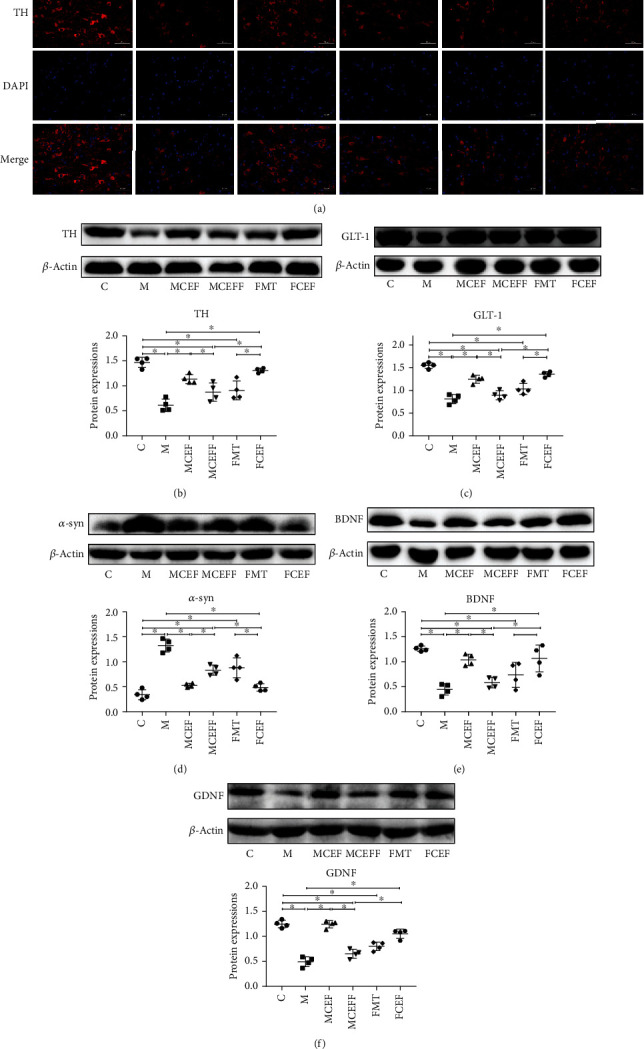
Ceftriaxone alleviated MPTP-induced neuropathologic changes in PD mice. (a) Ceftriaxone alleviated the reduction of dopamine neurons on mouse brain induced by MPTP (IF staining of substantia nigra). Western blotting of TH (b), GLT-1 (c), *α*-syn (d), BDNF (e), and GDNF (f) expression in substantia nigra, *β*-actin was used as an internal control. C group (*N* = 4), control group; M group (*N* = 4), MPTP group; MCEF group (*N* = 4), MPTP + ceftriaxone group; MCEFF group (*N* = 4), MPTP + ceftriaxone + fecal microbiota transplantation group; FMT group (*N* = 4), fecal microbiota transplantation group, FCEF group (*N* = 4), fecal microbiota transplantation group + ceftriaxone group. Data are presented as means ± SD. One-way repeated-measures ANOVA with Tukey's test for multiple comparisons (b–f, respectively); ^∗^*p* < 0.05, ^∗∗^*p* < 0.01. CEF: ceftriaxone; MPTP: 1-methyl-4-phenyl-1, 2, 3, 6-tetra-hydropyridine.

To evaluate the effect of ceftriaxone on PD mice neuropathologically, immunofluorescence was used to detect TH expression in the substantia nigra of mice. MPTP significantly reduced the TH expression in the substantia nigra of mice in the M group (Figure 2(a)). Based on Western blotting, MPTP treatment significantly decreased the expression of TH, GLT-1, and neurotrophic factors, and significantly increased the expression of *α*-syn compared with the C group (Figures 2(b)–2(f)). The transplantation of microbiota from PD mice worsened the neuropathological characteristics in the MCEFF and FMT groups, while the use of ceftriaxone greatly enhanced the expression of TH, GLT-1, and neurotrophic factors and reduced the expression of *α*-syn (Figure 2).

**Figure 3:**
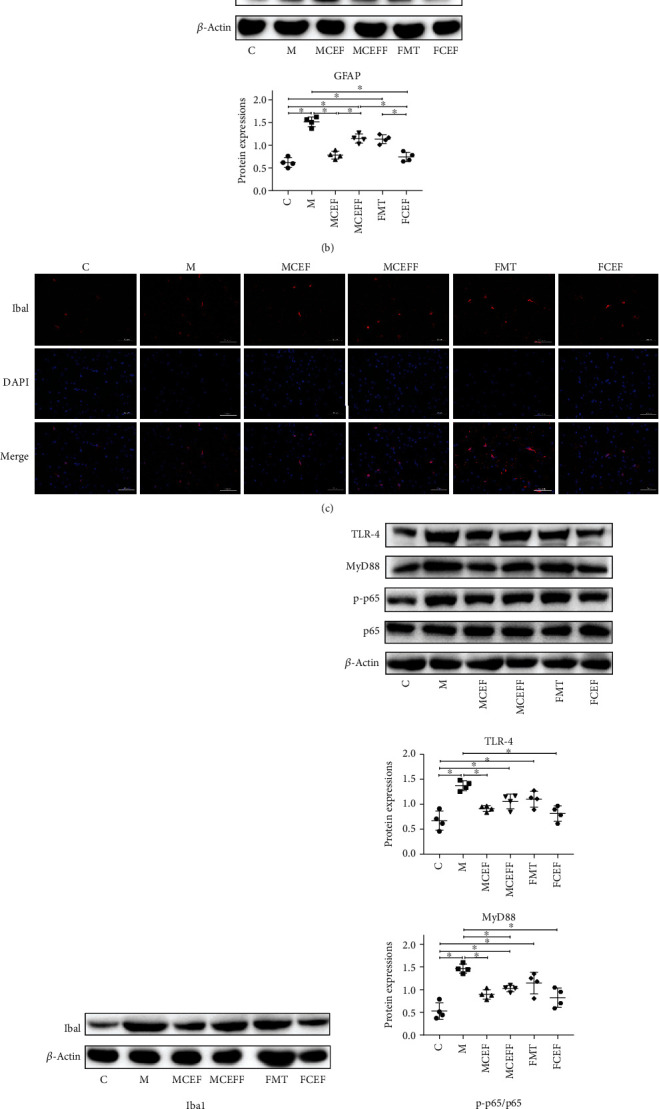
Ceftriaxone alleviates MPTP-induced neuroinflammation. (a) Ceftriaxone decreased the expression of GFAP on mouse brain induced by MPTP (IF staining of substantia nigra). (b) Western blotting of GFAP expression in substantia nigra, *β*-actin was used as an internal control. (c) Ceftriaxone decreased the expression of Iba1 on mouse brain induced by MPTP (IF staining of substantia nigra). (d) Western blotting of Iba1 expression in substantia nigra, *β*-actin was used as an internal control. (e) Western blotting of TLR-4, MyD88, p-NF*κ*B, and NF*κ*B expression in substantia nigra, *β*-actin was used as an internal control. C group (*N* = 4), control group; M group (*N* = 4), MPTP group; MCEF group (*N* = 4), MPTP + ceftriaxone group; MCEFF group (*N* = 4), MPTP + ceftriaxone + fecal microbiota transplantation group; FMT group (*N* = 4), fecal microbiota transplantation group, FCEF group (*N* = 4), fecal microbiota transplantation group + ceftriaxone group. Data are presented as means ± SD. One-way repeated-measures ANOVA with Tukey's test for multiple comparisons (b, d, e, respectively); ^∗^*p* < 0.05, ^∗∗^*p* < 0.01. CEF: ceftriaxone; MPTP: 1-methyl-4-phenyl-1, 2, 3, 6-tetra-hydropyridine.

We further studied the effect of ceftriaxone on astrocytes and microglia; ceftriaxone significantly reduced the activation of glia via downregulating the expression of GFAP (a marker of astrocytes) and Iba1 (a marker of microglia) in the substantia nigra of mice (Figures 3(a)–3(d)). In addition, this drug reduced inflammation by lowering the expression of TLR4, MyD88, and p-p65 in the TLR4/NF-*κ*B inflammatory pathway in the substantia nigra of mice (Figure 3(e)). Transplantation of microbiota from PD mice enhanced the activation of glia and increased the levels of TLR4/NF-*κ*B inflammatory proteins, and the use of ceftriaxone reversed these changes (Figure 3).

### 3.3. Effect of Ceftriaxone on the Intestinal Microbiota of MPTP-Induced PD Mice

**Figure 4:**
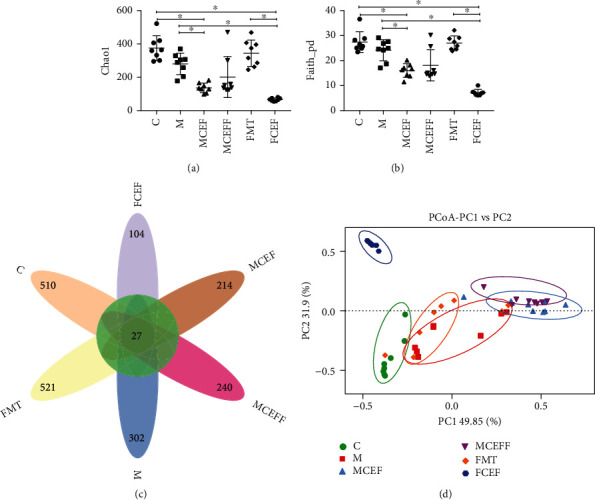
Gut microbial dysbiosis in PD mice is reduced by ceftriaxone. (a) The Chao1 index. (b) The Faith_pd index. (c) Petal map representation of OTUs. (d) PCoA of *β* diversity index. C group (*N* = 8), control group; M group (*N* = 8), MPTP group; MCEF group (*N* = 8), MPTP + ceftriaxone group; MCEFF group (*N* = 8), MPTP + ceftriaxone + fecal microbiota transplantation group; FMT group (*N* = 8), fecal microbiota transplantation group, FCEF group (*N* = 8), fecal microbiota transplantation group + ceftriaxone group. Data are presented as means ± SD. One-way repeated-measures ANOVA with Tukey's test for multiple comparisons (a, b, respectively); ^∗^*p* < 0.05, ^∗∗^*p* < 0.01. CEF: ceftriaxone; MPTP: 1-methyl-4-phenyl-1, 2, 3, 6-tetra-hydropyridine.

The intestinal microbiota plays a key role in the development and treatment of PD through the brain-gut axis [9]. Therefore, 16S rDNA amplicon sequencing was used to evaluate the effect of ceftriaxone on the intestinal microbiota of PD mice. The Chao1 index and Faith_pd index showed that there was no obvious difference between the C and M groups, while the use of ceftriaxone significantly reduced the *α*-diversity in the MCEF, MCEFF, and FCEF groups (Figures 4(a) and 4(b)). The Venn results revealed 27 common OTUs in all groups, and the number of unique OTUs in the C, M, MCEF, MCEFF, FMT, and FCEF groups is 510, 302, 214, 240, 521, and 104, respectively (Figure 4(c)). PCoA analysis indicated that the samples in the M group were far from samples in the C group, and the transplantation of faecal microbiota from PD mice (MCEFF and FMT groups) significantly changed the microbial diversity compared with the C group. Moreover, the use of ceftriaxone (MCEF, MCEFF, and FCEF groups) greatly changed the microbial diversity compared with the C group (Figure 4(d)), even though these mice had obvious changes in behaviour and neuroinflammation caused by MPTP and PD faecal microbiota transplantation.

**Figure 5:**
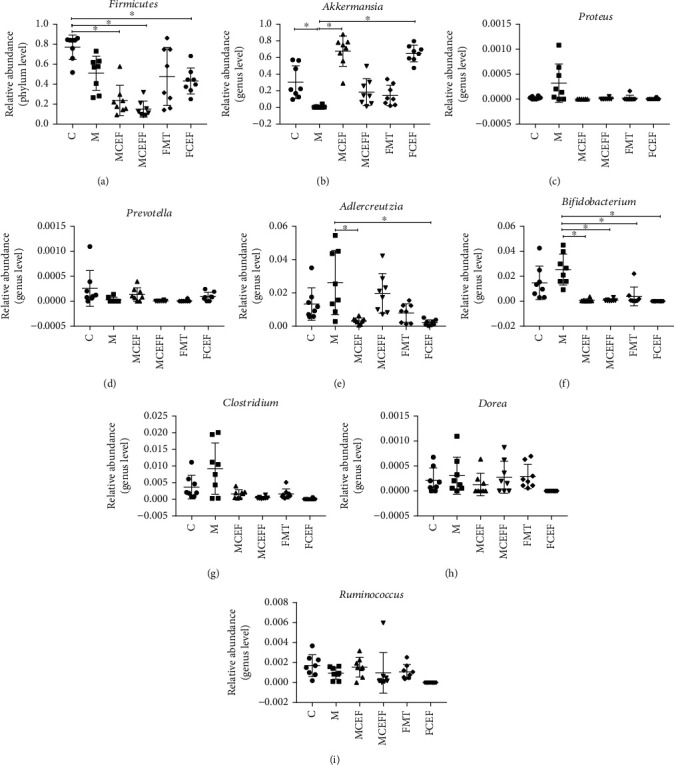
Effect of ceftriaxone on the relative abundance of gut microbial at phylum and genus level in feces of mice. The relative abundance of *Firmicutes* (a), *Akkermansia* (b), *Proteus* (c), *Prevotella* (d), *Adlercreutzia* (e), *Bifidobacterium* (f), *Clostridium* (g), *Dorea* (h), and *Ruminococcus* (i), in feces of PD mice. C group (*N* = 8), control group; M group (*N* = 8), MPTP group; MCEF group (*N* = 8), MPTP + ceftriaxone group; MCEFF group (*N* = 8), MPTP + ceftriaxone + fecal microbiota transplantation group; FMT group (*N* = 8), fecal microbiota transplantation group, FCEF group (*N* = 8), fecal microbiota transplantation group + ceftriaxone group. Data are presented as means ± SD. One-way repeated-measures ANOVA with Tukey's test for multiple comparisons (a–i, respectively); ^∗^*p* < 0.05,^∗∗^*p* < 0.001. CEF: ceftriaxone; MPTP: 1-methyl-4-phenyl-1, 2, 3, 6-tetra-hydropyridine.

Finally, we compared the relative abundance of probiotics and pathogens closely related to PD (Figure 5). MPTP reduced the abundance of the phylum Firmicutes and the genera *Akkermansia*, *Prevotella*, and *Ruminococcus* and markedly enhanced the abundance of the genera *Proteus*, *Adlercreutzia*, *Bifidobacterium*, *Clostridium*, and *Dorea* in the M group compared with the C group. Ceftriaxone treatment reduced the abundance of the genera *Bifidobacterium* and *Proteus* (*p* < 0.01) and increased the abundance of the probiotic genera *Akkermansia* and *Prevotella* (Figure 5).

### 3.4. Ceftriaxone Regulated Intestinal Tight Junction Proteins and Inhibited Intestinal Inflammation and Systemic Inflammation Caused by MPTP

**Figure 6:**
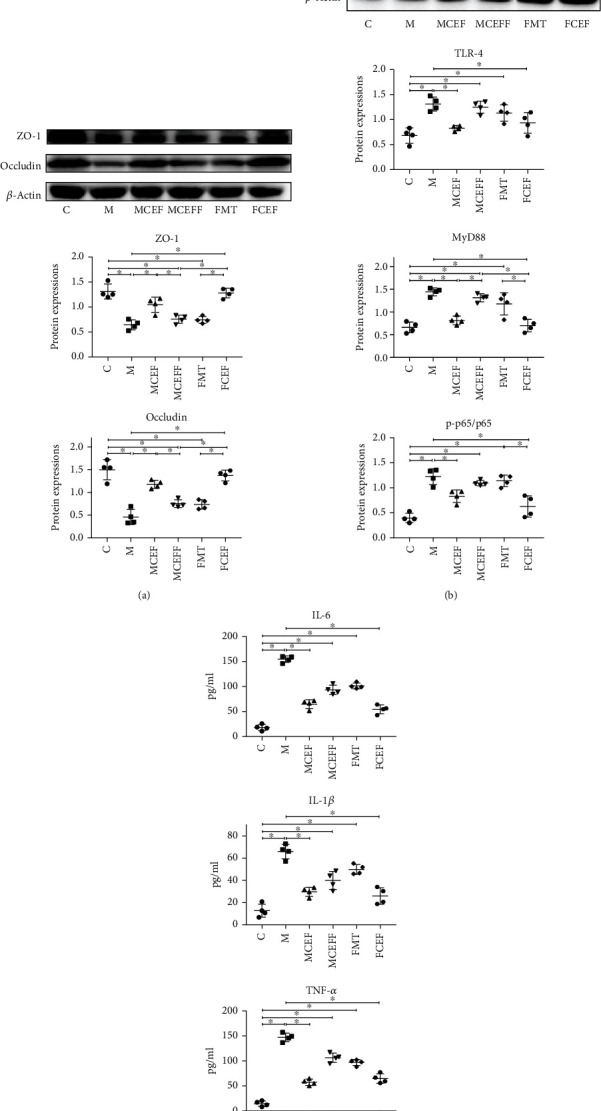
Ceftriaxone regulated intestinal tight junction proteins and inhibited intestinal inflammation and systemic inflammation caused by MPTP. (a) Western blot analysis of permeation-related proteins ZO-1, Occludin expression in the colon of mice, *β*-actin was used as an internal control. (b) Western blotting of TLR-4, MyD88, p-NF*κ*B, and NF*κ*B expression in in the colon of mice, *β*-actin was used as an internal control. (c) The expression of inflammatory cytokines TNF-*α* IL-6 and IL-1*β* in protein levels was detected by ELISA. C group (*N* = 4), control group; M group (*N* = 4), MPTP group; MCEF group (*N* = 4), MPTP + ceftriaxone group; MCEFF group (*N* = 4), MPTP + ceftriaxone + fecal microbiota transplantation group; FMT group (*N* = 4), fecal microbiota transplantation group, FCEF group (*N* = 4), fecal microbiota transplantation group + ceftriaxone group. Data are presented as means ± SD. One-way repeated-measures ANOVA with Tukey's test for multiple comparisons (a–c, respectively); ^∗^*p* < 0.05, ^∗∗^*p* < 0.01. CEF: ceftriaxone; MPTP: 1-methyl-4-phenyl-1, 2, 3, 6-tetra-hydropyridine.

To further investigate the role of intestinal microbiota in the brain-gut axis, we assessed the expression of intestinal tight-junction-associated proteins and proteins in the TLR4/NF-*κ*B inflammatory pathway in the colon (Figure 6). MPTP significantly reduced the expression of ZO-1 and occludin (Figure 6(a)); increased the expression of TLR4, MyD88, and p-p65 in the M group compared with the C group (Figure 6(b)); and also promoted the expression of inflammatory cytokines IL-6 (M vs.C = 154.7 pg/ml vs.17.94 pg/ml; *p* < 0.01), IL-1*β* (M vs.C = 65.93 pg/ml vs.12.74 pg/ml; *p* < 0.01), and TNF-*α* (M vs.C = 147.2 pg/ml vs.14.47 pg/ml; *p* < 0.01) in the serum of mice (Figure 6(c)). The use of ceftriaxone significantly reduced the colonic inflammatory proteins and systemic inflammatory cytokines caused by MPTP and faecal microbiota transplantation from PD mice and greatly enhanced the expression of intestinal tight-junction-associated proteins.

## 4. Discussion

PD is a common degenerative disease of the central nervous system; the number of patients with PD is predicted to exceed 17.5 million by 2040 [1]. At present, there is no specific medicine to cure PD [19]. Therefore, considering the difficulties of new drug development, exploiting new features of old drugs is a good choice [13]. Ceftriaxone is a broad-spectrum antibiotic that is used commonly in the clinic to treat infections of the respiratory tract and urinary system [16]; it exhibits a neuroprotective effect in addition to its antibacterial effect in PD [15, 20] and AD [21]. However, little work has been done to explore the effect of ceftriaxone on the intestinal microbiota in PD.

In this study, a mouse model of subacute PD was used to evaluate the effect of ceftriaxone on PD, and faecal microbiota transplantation was performed (faecal microbiota from PD mice were transplanted into normal mice) to evaluate whether ceftriaxone plays an important role in regulating the intestinal microbiota of PD mice. MPTP caused significant motor dysfunction and decreased the exploratory ability of mice, and the mice receiving faecal microbiota from PD mice also showed similar behaviours. Intraperitoneal injection of ceftriaxone reversed the behavioural changes caused by MPTP and PD faecal microbiota. These results are consistent with previous reports that ceftriaxone could improve the motor dysfunction of PD [20, 22].

The dopaminergic and glutamatergic systems in the brain work together to regulate motor and cognitive functions [23]. TH is the rate-limiting enzyme that participates in dopamine synthesis, and when it is reduced to a certain threshold, it will cause the motor symptoms of PD [24]. Meanwhile, the excitotoxicity of glutamate plays an important role in the pathogenesis of PD [25]. Glutamate in the brain is mainly cleared by GLT-1, but GLT-1 shows dysfunction or decreased expression in patients with neurodegenerative diseases such as PD [26], AD [27], or ALS [17]. Ceftriaxone reportedly reduced the loss of dopaminergic neurons in PD patients by increasing GLT-1 expression [23], and our results also confirmed that ceftriaxone could increase TH and GLT-1 expression in mouse substantia nigra. In addition, BDNF and GDNF are dopamine-active neurotrophic proteins that can promote the survival of dopaminergic neurons and play an important role in neurite growth [28]. Our results showed that ceftriaxone restored the expression of BDNF and GDNF in MPTP-induced PD mice, a finding that is consistent with the results of Kaur and Prakash [15]. In summary, these results indicate that ceftriaxone could increase the expression of TH, GLT-1, and neurotrophic factors, thereby improving the motor symptoms of PD mice.

In recent years, increasing evidence has confirmed that the neuroinflammatory response is involved in the pathogenesis of PD. Neuroinflammation is characterised by the activation of microglia and astrocytes [29]. Microglia is the inherent immune effector cells in the central nervous system [30, 31]. Under normal circumstances, microglia are in a resting state. When brain tissue is damaged or stimulated by *α*-syn or bacteria and their metabolites in the brain, TLR4 is activated on microglia, and then, the NF-*κ*B pathway is activated to promote the secretion of proinflammatory factors such as IL-6, IL-1*β*, and TNF-*α*; the ultimate outcome is the death of neurons [30, 32]. The signalling pathway mediated by TLR4 plays an important role in PD [33], and knockout of the *Tlr4* gene reduces rotenone-induced motor dysfunction, neuroinflammation, and corresponding neuropathological changes in PD mice [33]. In this study, we found that ceftriaxone reduced the expression of GFAP and Iba1 and downregulated the expression levels of TLR4, MyD88, and p-p65, all part of the inflammatory pathway.

To further explore the effect of ceftriaxone on the intestinal microbes of PD mice, 16S rDNA amplicon sequencing was used to detect changes in gut microbes in the mice. Through the Chao1 index and Faith_pd index, we found that MPTP treatment reduced the *α*-diversity of the intestinal microbiota. Although ceftriaxone treatment also significantly reduced the *α*-diversity of the intestinal microbiota, when we analysed microbiota changes at the phylum and genus levels, we found that the abundance of the genera *Akkermansia* and *Prevotella* was increased in the ceftriaxone-treated groups. Many studies have shown that *Akkermansia* can effectively improve symptoms in mouse models of diseases such as ALS [34], progeria [35], and chronic colitis [36] by promoting healing and recovery of intestinal mucosal layer and reducing the expression of proinflammatory cytokines such as TNF-*α* and interferon gamma (IFN-*γ*) in the colon. *Prevotella* has been reported to be correlated negatively with PD [37] and autism spectrum disorder (ASD) [38]. In addition, MPTP administration increased the abundance of the genera *Proteus*, *Adlercreutzia*, *Bifidobacterium*, and *Clostridium*, as well as the bacterium *Proteus mirabilis* that may trigger neuroinflammation and lead to pathological changes related to PD by its metabolites (such as lipopolysaccharide) [39]. Although studies have shown that *Lactobacillus acidophilus* and *Bifidobacterium infantis* can improve abdominal pain and bloating in PD patients [40], the results reported by Petrov et al. [37] and our research consistently showed that the abundance of *Bifidobacterium* in PD is increased.

The functions of the intestinal mucosal barrier are related to the intestinal microbiota, whose dysbiosis can lead to decreased expression of intestinal tight-junction-associated proteins. This reduction allows the entry of pathogenic bacteria and their metabolites into the blood and increases inflammatory factors such as IL-6, IL-1*β*, and TNF-*α* [36]. There is an increase in proinflammatory microorganisms in the intestinal microbiota of PD patients [41], and proinflammatory cytokines could play a role in the nonmotor symptoms of PD [42]. In our study, ceftriaxone therapy restored the MPTP-induced decrease in intestinal tight-junction-associated proteins, reducing TLR4-mediated intestinal inflammation as well as serum IL-6, IL-1*β*, and TNF-*α* expression.

## 5. Conclusion

We conclude that ceftriaxone regulates the intestinal microbiota of PD mice and alleviates the motor dysfunction and decreased exploratory ability. It also plays an important role in increasing the integrity of the intestinal barrier, reducing the inflammation of the colon and the brain, and has a neuroprotective effect (Supplemental Figure 1). Ceftriaxone could be a potential treatment for PD, but we only examined the short-term effects of ceftriaxone on PD through the establishment of an MPTP-induced PD mouse model. Further work is needed to determine the long-term effects of ceftriaxone by using transgenic mice. In addition, because ceftriaxone drug resistance could appear after long-term use, the dose and frequency of ceftriaxone should be explored.

## Data Availability

The datasets used and/or analyzed during the present study are available from the corresponding author on reasonable request.

## Supplementary Materials

Supplementary Materials Supplemental Figure1: the graphical abstract of our study. Ceftriaxone can alleviate the motor dysfunction and decreased exploration ability. In addition, it also plays an important role in increasing the integrity of the intestinal barrier, reducing the inflammation of the colon and brain, and has the neuroprotective effect, and its neuroprotective effect may be through regulating inflammation and intestinal microbiota.
