# Correction to “Neuroprotective Effect of Ceftriaxone on MPTP‐Induced Parkinson’s Disease Mouse Model by Regulating Inflammation and Intestinal Microbiota”

**DOI:** 10.1155/omcl/9853756

**Published:** 2026-02-18

X. Zhou, J. Lu, K. Wei, J. Wei, P. Tian, M. Yue, Y. Wang, D. Hong, F. Li, B. Wang, T. Chen, and X. Wang, “Neuroprotective Effect of Ceftriaxone on MPTP‐Induced Parkinson’s Disease Mouse Model by Regulating Inflammation and Intestinal Microbiota” *Oxidative Medicine and Cellular Longevity*, vol. 2021 (2021). https://doi.org/10.1155/2021/9424582.

In the article titled “Neuroprotective Effect of Ceftriaxone on MPTP‐Induced Parkinson’s Disease Mouse Model by Regulating Inflammation and Intestinal Microbiota,” there was an error in Figure 2a. More specifically, the panels depicting the immunofluorescence staining of the FMT group share overlapping features with the panels representing the M group. These errors were introduced by the authors during figure assembly and should be corrected as follows:

**Figure 2:**
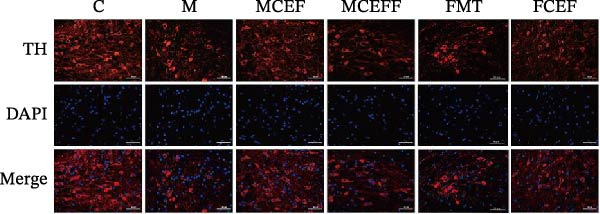
Ceftriaxone alleviated MPTP‐induced neuropathologic changes in PD mice. (a) Ceftriaxone alleviated the reduction of dopamine neurons on mouse brain induced by MPTP (IF staining of substantia nigra). Western blotting of TH (b), GLT‐1 (c), α‐syn (d), BDNF (e), and GDNF (f) expression in substantia nigra, β‐actin was used as an internal control. C group (*N* = 4), control group; M group (*N* = 4), MPTP group; MCEF group (*N* = 4), MPTP + ceftriaxone group; MCEFF group (*N* = 4), MPTP + ceftriaxone + fecal microbiota transplantation group; FMT group (*N* = 4), fecal microbiota transplantation group, FCEF group (*N* = 4), fecal microbiota transplantation group + ceftriaxone group. Data are presented as means ± SD. One‐way repeated‐measures ANOVA with Tukey’s test for multiple comparisons (b–f, respectively);  ^∗^ *p*  < 0.05,  ^∗^^∗^ *p*  < 0.01. CEF: ceftriaxone; MPTP: 1‐methyl‐4‐phenyl‐1, 2, 3, 6‐tetra‐hydropyridine.

We apologize for these errors.

