# Supplementary material for: Neuroprotective Effect of Ceftriaxone on MPTP-Induced Parkinson's Disease Mouse Model by Regulating Inflammation and Intestinal Microbiota
Source: Oxid Med Cell Longev. 2021 Dec 13;2021:9424582. doi: 10.1155/2021/9424582 (PMC8687851; doi:10.1155/2021/9424582)
Supplement: Supplementary Materials — Supplemental Figure1: the graphical abstract of our study. Ceftriaxone can alleviate the motor dysfunction and decreased exploration ability. In addition, it also plays an important role in increasing the integrity of the intestinal barrier, reducing the inflammation of the colon and brain, and has the neuroprotective effect, and its neuroprotective effect may be through regulating inflammation and intestinal microbiota. [file 9424582.f1.pdf]

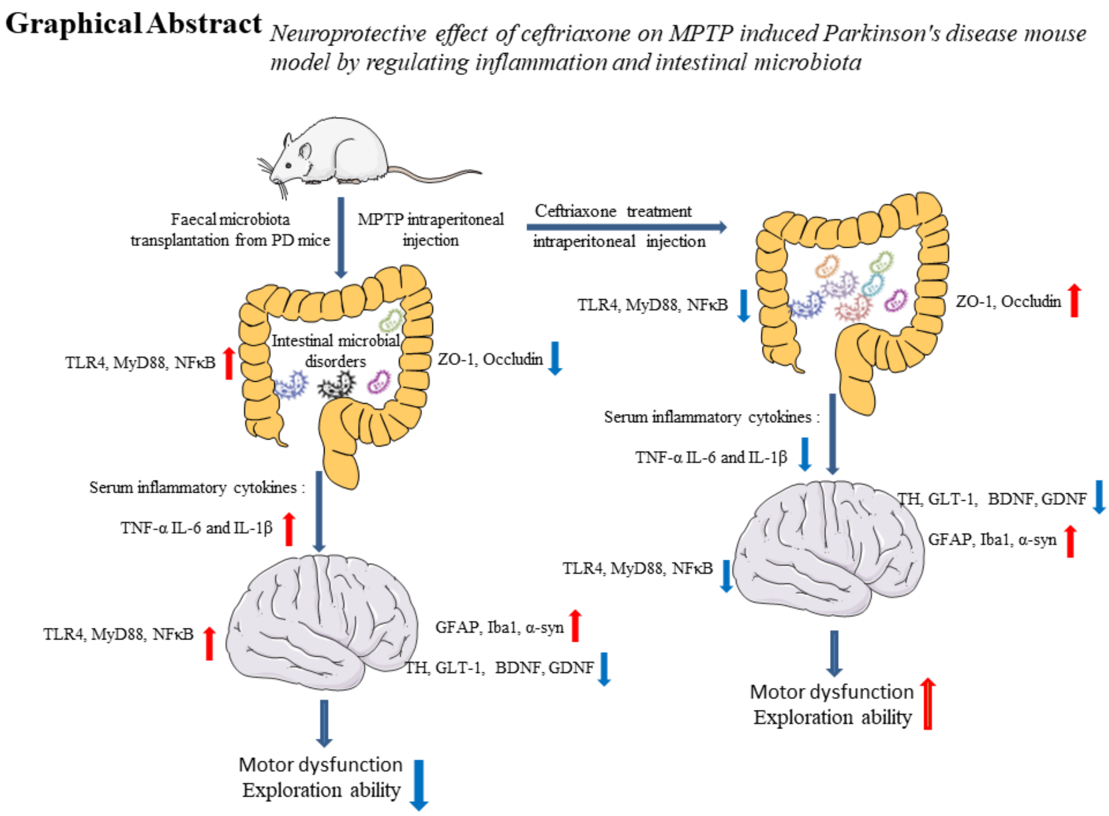

Supplemental Figure 1: The graphical abstract of our study. Ceftriaxone can alleviate the motor dysfunction and decreased exploration ability. In addition, it also plays an important role in increasing the integrity of the intestinal barrier, reducing the inflammation of the colon and brain, and has the neuroprotective effect, and its neuroprotective effect may be through regulating inflammation and intestinal microbiota.
